# Core Nanoparticle Engineering for Narrower and More Intense Band-Edge Emission from AgInS_2_/GaS_x_ Core/Shell Quantum Dots

**DOI:** 10.3390/nano9121763

**Published:** 2019-12-11

**Authors:** Watcharaporn Hoisang, Taro Uematsu, Takahisa Yamamoto, Tsukasa Torimoto, Susumu Kuwabata

**Affiliations:** 1 Department of Applied Chemistry, Graduate School of Engineering, Osaka University, 2-1 Yamada-oka, Suita, Osaka 565-0871, Japan; hwatcharaporn@chem.eng.osaka-u.ac.jp (W.H.); t-uematsu@chem.eng.osaka-u.ac.jp (T.U.); 2 Department of Quantum Engineering, Graduate School of Engineering, Nagoya University, Chikusa-ku, Nagoya 464-8603, Japan; yamamoto.takahisa@material.nagoya-u.ac.jp; 3 Department of Crystalline Materials Science, Graduate School of Engineering, Nagoya University, Chikusa-ku, Nagoya 464-8603, Japan; torimoto@chembio.nagoya-u.ac.jp

**Keywords:** silver indium sulfide, I–III–VI semiconductor, quantum dots, dropwise injection method, band-edge emission

## Abstract

Highly luminescent silver indium sulfide (AgInS_2_) nanoparticles were synthesized by dropwise injection of a sulfur precursor solution into a cationic metal precursor solution. The two-step reaction including the formation of silver sulfide (Ag_2_S) nanoparticles as an intermediate and their conversion to AgInS_2_ nanoparticles, occurred during the dropwise injection. The crystal structure of the AgInS_2_ nanoparticles differed according to the temperature of the metal precursor solution. Specifically, the tetragonal crystal phase was obtained at 140 °C, and the orthorhombic crystal phase was obtained at 180 °C. Furthermore, when the AgInS_2_ nanoparticles were coated with a gallium sulfide (GaS_x_) shell, the nanoparticles with both crystal phases emitted a spectrally narrow luminescence, which originated from the band-edge transition of AgInS_2_. Tetragonal AgInS_2_ exhibited narrower band-edge emission (full width at half maximum, FWHM = 32.2 nm) and higher photoluminescence (PL) quantum yield (QY) (49.2%) than those of the orthorhombic AgInS_2_ nanoparticles (FWHM = 37.8 nm, QY = 33.3%). Additional surface passivation by alkylphosphine resulted in higher PL QY (72.3%) with a narrow spectral shape.

## 1. Introduction

Luminescent semiconductor nanoparticles, or quantum dots (QDs), have attracted attention over the past two decades due to their unique optical and electronic properties owing to the quantum size effect [1,2]. The optical properties of QDs represented by tunable emission colors on the basis of size and/or composition, high absorption coefficient, and high photoluminescence (PL) quantum yield (QY) are the characteristics of these materials [3]. For instance, chalcogenide binary semiconductor QDs (e.g., CdS, CdSe, and PbS) have been investigated for the use in several optoelectronic and magnetic applications such as wavelength conversion for display devices, light-emitting diodes, chemical sensors, and other spintronic devices [4,5,6,7,8]. However, the high intrinsic toxicity of cadmium (Cd) and lead (Pb) in these QDs limits their potential applications; therefore, alternative materials are needed.

Cadmium-free ternary I–III–VI semiconductor QDs (e.g., AgInS_2_ and CuInS_2_) have been intensively investigated to replace cadmium-containing QDs. These cadmium-free QDs possess band gap energies in the visible region as well as large optical absorption coefficients, which are characteristic for direct semiconductors [9,10,11,12,13]. Among these QDs, silver indium sulfide (AgInS_2_) nanoparticles (NPs) with a band gap energy of 1.87 eV have attracted increasing attention [14,15]. A universal challenge during the synthesis of AgInS_2_ NPs is to balance the reactivity of two cationic precursors against one anionic precursor [10,16]. In the past, the thermal decomposition of a single molecular precursor was used to avoid the reactivity problem, which resulted in a high (~50%) PL QY for AgInS_2_-ZnS NPs [17,18,19]. The limitations of this method are the necessity of designing a molecular precursor for each composition and the difficulty of controlling the particle size and shape due to the complexity of the decomposition process [20]. Alternatively, the reactions of the cation mixture with sulfur-containing species at higher temperatures are common. A typical example uses silver nitrate (AgNO_3_), indium acetate (In(OAc)_3_), and 1-dodecanethiol (DDT) dissolved in 1-octadecene (ODE), which were heated until DDT reacted with metal cations to generate AgInS_2_ NPs [21]. Although the “heating up” approach achieved better PL QY, it was still difficult to regulate the particle size because the growth process mainly occurred by Ostwald ripening [22,23]. At the same time, the method of particle size control by the rapid injection of precursors into a hot solvent, which has been commonly used for binary semiconductor QDs, was also adopted for the AgInS_2_ NPs synthesis and achieved good size distribution with a PL QY as high as 59% [24,25,26]. Although the control over particle size and composition was achieved, none of these attempts generated a narrow band-edge emission corresponding to common II–VI semiconductor QDs.

Recently, we have successfully demonstrated a narrow band-edge PL from AgInS_2_ NPs by coating them with a gallium sulfide (GaS_x_) shell to passivate the surface of AgInS_2_ NPs [27,28]. Although the surface passivation with an amorphous GaS_x_ is essential for generating band-edge emission, the PL QY was considerably affected by the properties of the AgInS_2_ core NPs. Specifically, the highest QY value of the band-edge emission (56% after the surface passivation with tri-*n*-octylphosphine) was obtained when using the AgInS_2_ core particles with a 4-nm diameter and tetragonal phase. These core NPs showed a high PL QY (64%) by themselves, although the spectral shape originated from a broad defect emission. Therefore, we determined that the core and surface of NPs should be defect-free. These preferred AgInS_2_ cores were obtained by a two-step reaction using small silver sulfide (Ag_2_S) NPs as intermediate species.

In this study, the dropwise addition of sulfur sources in liquid was adopted instead of adding solid thiourea all at once. This approach was adopted because the control of the reaction speed for both Ag_2_S nucleation and conversion to AgInS_2_ is essential to obtain better cores. The speed of injection and reaction temperature were changed to control the particle size and crystal structures of the AgInS_2_ cores. The GaS_x_ shell coating on the prepared AgInS_2_ NPs exhibited band-edge PL, which was still narrower than that in our previous report. The highest PL QY of the as-prepared AgInS_2_/GaS_x_ core/shell QDs was ~50%, and the value increased to 70% after the surface treatment with tri-*n*-octylphosphine.

## 2. Materials and Methods

### 2.1. Materials

Silver acetate (Ag(OAc), 99.9%) and 1-dodecanethiol (DDT, 98.0%) were purchased from Fujifilm Wako Pure Chemical, Japan. Indium acetate (In(OAc)_3_, 99.9%) and gallium acetylacetonate (Ga(acac)_3_, 99.9%) were obtained from Sigma-Aldrich, USA. 1,3-dimethylthiourea (DMTU, >97.0%) and tri-*n*-octylphosphine (TOP, >85%) were supplied by Tokyo Chemical Industry, Japan. The above-mentioned chemicals were used as received without further purification. Oleylamine (OAm, >50%) was purchased from Fujifilm Wako Pure Chemical and then purified by vacuum distillation in the presence of calcium hydride.

### 2.2. Instrumental

The temperature of reaction mixtures was measured directly by a thermocouple and was controlled by a heating mantle equipped with a PID controller. UV–Vis absorption and PL spectra were recorded using a double beam UV–Vis spectrophotometer (JASCO, Japan, V-670) and a fluorospectrometer (JASCO, Japan, FP-8600). Photoluminescence QY was measured by a diode-array spectrometer equipped with an integrating sphere (Hamamatsu, Japan, PMA12). PL lifetimes were recorded with a time-correlated single-photon counting set-up (Hamamatsu, Japan, Quantaurus-Tau). The size and morphology of NPs were observed by a transmission electron microscopy (TEM) instrument (Hitachi, Japan, H-7650) at an acceleration voltage of 100 kV. High-resolution TEM (HRTEM) and high-angle annular dark-field scanning transmission electron microscopy (HAADF-STEM) images were taken by a Cs-corrected HR-STEM (JEOL, Japan, JEM-ARM-200F) at an acceleration voltage of 200 kV. Carbon-coated copper TEM grids (Oken-shoji, Japan, HRC-C10) were used for the preparation of TEM samples. X-ray powder diffraction (XRD) patterns were collected by a powder x-ray diffractometer (Rigaku, Japan, SmartLab) equipped with a parallel beam/parallel slit analyzer. The composition of NPs was analyzed by either a scanning electron microscopy (SEM) instrument (Hitachi, Japan, S-3400N) equipped with an energy-dispersive x-ray (EDX) detector (AMETEK, USA, EDAX X1) or an inductively coupled plasma atomic emission spectroscopy (ICP-AES) instrument (Shimadzu, Japan, ICPS-7510).

### 2.3. Synthesis of AgInS_2_ Core Nanoparticles by the Immediate Injection Method

Ag(OAc) (0.4 mmol), In(OAc)_3_ (0.4 mmol), and DDT (1.2 mmol) were mixed with OAm (8 mL) in a 50 mL two-necked round bottom flask. The mixture was stirred under Ar and heated to 120 °C. 1,3-dimethylthiourea (0.8 mmol) dissolved in OAm (1 mL) was injected into the metal precursors mixture all at once. The temperature of the solution was elevated to 140 °C and kept at this level for 30 min to grow NPs. After the reaction was completed, the solution was cooled to room temperature and centrifuged to remove large particles. For purification, the OAm-capped AgInS_2_ NPs were precipitated by the addition of excess methanol and dispersed in chloroform.

### 2.4. Synthesis of AgInS_2_ Core NPs by the Dropwise Injection Method

Ag(OAc) (0.4 mmol), In(OAc)_3_ (0.4 mmol), and DDT (1.2 mmol) were mixed with OAm (8 mL) in a two-necked round bottom flask. The mixture was heated to 140 °C under Ar, followed by the dropwise injection of 0.4 M DMTU dissolved in OAm using a syringe pump. The flow rate of the injection was varied in the range of 2–16 mL/h, but the total amount was set to 2 mL. The solution temperature was maintained at 140 °C for 30 min after the injection was completed. The solution was then cooled to room temperature, and the purification of the NPs was performed using the above-mentioned approach.

### 2.5. GaS_x_ Shell Formation on AgInS_2_ Core NPs

AgInS_2_/GaS_x_ core/shell NPs were synthesized according the method described in our previous report with slight modifications [27]. Typically, AgInS_2_ core NPs (30 nmol in terms of particles), Ga(acac)_3_ (0.1 mmol), and DMTU (0.1 mmol) were mixed with OAm (7 mL), and the solution was degassed under vacuum. Under an Ar atmosphere, the solution was rapidly heated to 230 °C, and then the temperature was increased to 280 °C at a rate of 2 °C/min. The color of the solution changed from red to orange, which indicated that the GaS_x_ shell was formed on the AgInS_2_ core surface. The reaction mixture was allowed to cool to room temperature, and the core/shell NPs were purified by the addition of methanol to precipitate the NPs, followed by centrifugation and dispersion in chloroform. Finally, TOP was added to the core/shell NP solutions 1:1 to chloroform and the mixed solutions were maintained at room temperature to complete post-treatment (caution: TOP can be flammable if a small spill is wiped up with a Kimwipe).

## 3. Results and Discussion

### 3.1. Synthesis of High-Quality AgInS_2_ Quantum Dots

In our previous procedures for synthesizing AgInS_2_ cores, thiourea solid was put directly into the metal precursor solution. To better control particle size and composition during the synthesis of ternary NPs, it is essential to control the nucleation of intermediate Ag_2_S NPs. As thiourea is barely soluble in oleylamine and the lowest polarity solvent used for synthesizing NPs, an alternative sulfur source with similar reactivity, 1,3-dimethylthiourea (DMTU), was adopted. The AgInS_2_ core QDs were synthesized by introducing the oleylamine solution of DMTU into preheated metal precursor solutions. Two types of injection methods were attempted (i.e., immediate and dropwise injections). The immediate injection method is similar to the hot injection method, which is commonly used for synthesizing II–VI semiconductors. However, as a result of empirical optimization, the temperature at which the DMTU solution was injected, was considerably lower (120 °C) than that typically used for II–VI semiconductors (>200 °C). Another dropwise injection method followed the solid thiourea method that takes advantage of the slow dissolution of solid thiourea to release sulfur. In both cases, when the DMTU solution was injected, a pale-yellow solution (Ag–dodecanethiol complex) became orange, red, and finally dark brown, which indicated the formation of the Ag_2_S intermediate. The Ag_2_S intermediate was subsequently converted to AgInS_2_ by cation exchange or resource–host-type reactions [29,30].

**Figure 1:**
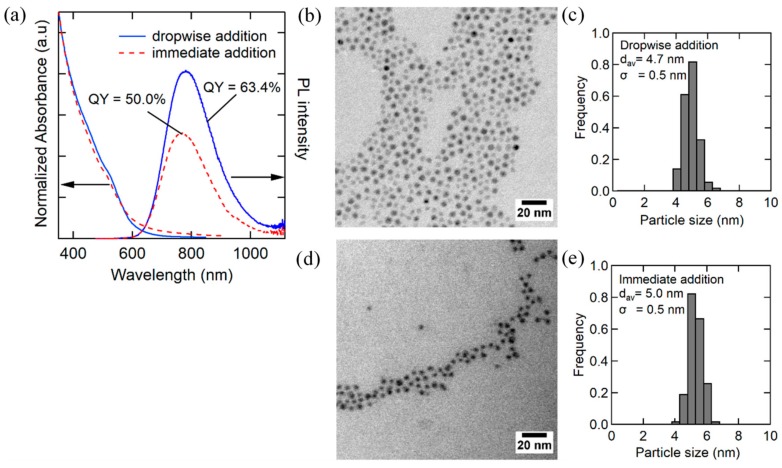
(**a**) UV–Vis absorption and photoluminescence (PL) spectra of AgInS_2_ core nanoparticles (NPs) synthesized by different modes for 1,3-dimethylthiourea (DMTU) addition; (red line) immediate injection and (blue line) dropwise injection at 4 mL/h to metal precursor solution heated at 140 °C. Transmission electron microscopy (TEM) images and the corresponding size distribution histograms of AgInS_2_ NPs prepared by (**b**,**c**) immediate and (**d**,**e**) dropwise injection.

**Figure 2:**
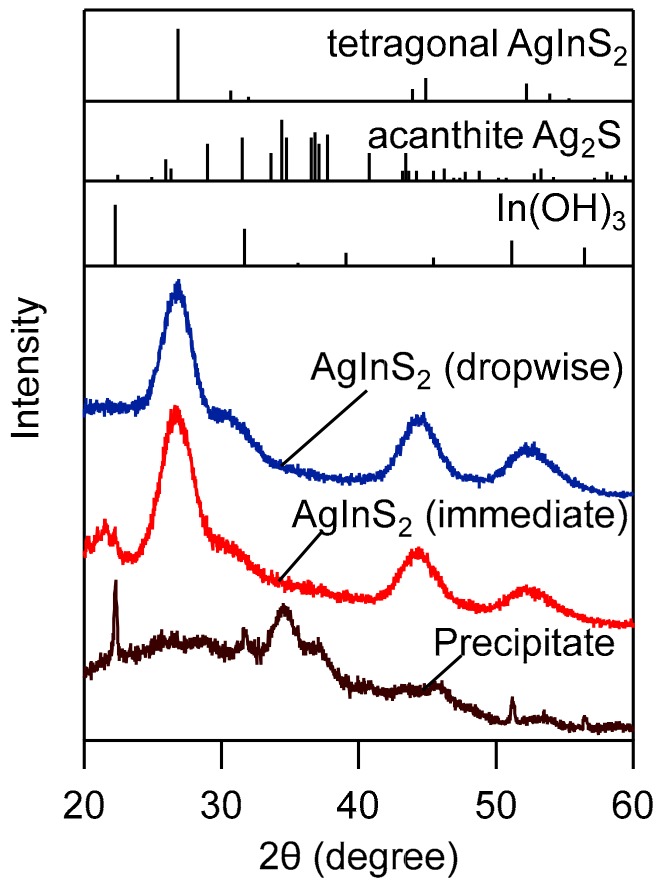
X-ray powder diffraction (XRD) patterns of AgInS_2_ NPs synthesized by different methods; (red line) immediate and (blue line) dropwise injection methods; 4 mL/h injection rate into a metal precursor solution that was heated to 140 °C; (brown line) large particles, which were separated after the reaction. XRD patterns of tetragonal-AgInS_2_ (ICSD 077-6632), acanthite Ag_2_S (ICSD 014-0072), and In(OH)_3_ (ICSD 085-1338) are presented as references.

Figure 1 shows the UV–Vis, PL spectra, and TEM images and size distribution histograms of AgInS_2_ QDs synthesized by the immediate injection and dropwise injection methods. In the immediate injection method, the reaction temperature was increased from 120 °C (injection temperature) to 140 °C to induce the step-by-step growth of Ag_2_S NP and conversion to AgInS_2_. On the other hand, in the dropwise injection method, the temperature of the metal precursor solution remained at 140 °C throughout the reaction, to which DMTU solution (2 mL) was slowly injected at a rate of 4 mL/h. After finishing the injection, the heating was continued for an additional 30 min to complete the conversion from Ag_2_S to AgInS_2_. After centrifugation to remove large insoluble particles, dark-red clear solutions were obtained, regardless of the injection methods. Both types of samples contained nearly monodisperse NPs. The average particle size was slightly smaller (4.6 nm) and PL QY was higher (63.4%) for the NPs synthesized by the dropwise injection method than those synthesized by the immediate injection method (average size = 5.0 nm and PL QY = 50%). The XRD patterns of both samples consisted of three broad peaks at 2θ = 26.8°, 44.8°, and 52.2°, which correspond to the (112), (204), and (312) planes of tetragonal AgInS_2_, respectively (Figure 2). However, the large particles removed by centrifugation exhibited small, broad XRD peaks, which are partly attributed to acanthite Ag_2_S and In(OH)_3_; the latter may have originated from indium acetylacetonate. Typically, in colloid synthesis, an immediate injection of reaction species is effective for the separation of nucleation and growth processes to obtain monodisperse NPs. The dropwise injection of precursors was adopted for the synthesis of NP shells to keep the concentration of reactive species low and to prevent nucleation. However, the particle size distribution of AgInS_2_ QDs synthesized by the two different methods was similar, and both cases resulted in a size distribution as narrow as σ = 0.5 nm. The effectiveness of the dropwise method in terms of size control and higher PL QY may be related to the unique two-step reaction mechanism during which AgInS_2_ NPs are formed through an intermediate Ag_2_S. In other words, Ag_2_S NPs or clusters needed to be quickly converted to silver indium sulfide after the formation, otherwise they become too large and precipitate out in the reaction solution.

The elemental compositions of AgInS_2_ QDs prepared by the immediate and dropwise injection methods were measured using SEM–EDX, and the results are shown in Figure S1 (Supplementary Materials). The atomic ratio of the sample prepared by the immediate injection of the sulfur source was Ag:In:S = 1:0.69:1.75, whereas Ag:In:S = 1:0.9:2.2 with a more stoichiometric ratio was obtained for the NPs prepared by the dropwise injection method. The conversion from Ag_2_S to AgInS_2_ was relatively slower than the formation of Ag_2_S. In addition, intermediate Ag_2_S NPs were easily overgrown at 140 °C, as predicted by the report that Ag_2_S nanoparticles can be produced at room temperature [30]. Therefore, a portion of the Ag_2_S NPs remained in solution with an imperfect conversion to AgInS_2_, which increased the silver content when a large amount of Ag_2_S NPs was produced by the immediate injection method.

### 3.2. Effect of Injection Rate on the Dropwise Injection Method

**Figure 3:**
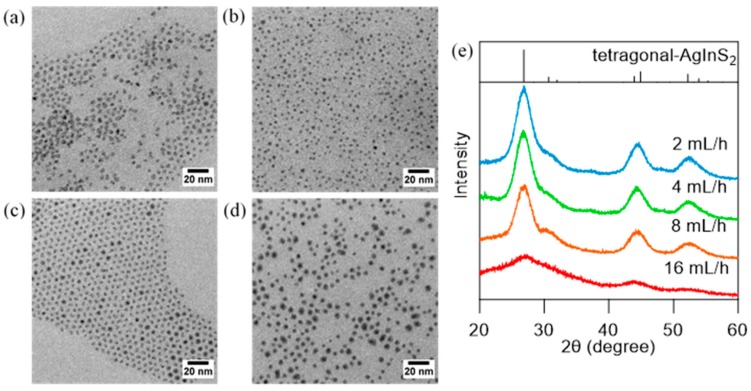
TEM images of AgInS_2_ NPs prepared by dropwise addition of DMTU at flow rate of (**a**) 2 mL/h, (**b**) 4 mL/h, (**c**) 8 mL/h, and (**d**) 16 mL/h. (**e**) XRD patterns of AgInS_2_ NPs prepared by dropwise injection method at various flow rate of DMTU addition: (blue) 2 mL/h, (green) 4 mL/h, (orange) 8 mL/h, and (red) 16 mL/h. XRD patterns of tetragonal-AgInS_2_ (ICSD 077–6632) is presented as reference.

The effect of the additional speed of DMTU was investigated by varying the injection rate of a syringe pump in the range of 2–16 mL/h. Figure 3 shows the TEM images and XRD patterns of AgInS_2_ QDs synthesized using different DMTU injection rates. Size histograms corresponding to each sample are presented in Figure S2. Highly monodispersed spherical NPs (*d*_av_ = 3–4 nm, σ < 0.5 nm) were observed for the samples prepared using 2, 4, and 8 mL/h flow rates. The sample prepared at 16 mL/h was slightly large and polydisperse (4.6 ± 0.7 nm), while the injection rate of 4 mL/h produced the smallest monodisperse AgInS_2_ NPs (*d*_av_ = 3.2 nm, σ = 0.3 nm). A further decrease in the injection rate (2 mL/h) increased the contribution of the Oswald ripening, which increased polydispersity. The XRD patterns showed a good agreement with those of tetragonal AgInS_2_, except for the samples prepared at 16 mL/h (Figure 3e). Furthermore, elemental analyses of each sample revealed a 1:1 inclusion of Ag:In except for the sample prepared at 16 mL/h (Ag:In = 1:0.2), which suggests that large spherical objects observed in Figure 3d primarily consisted of unconverted Ag_2_S NPs (Table S1).

**Figure 4:**
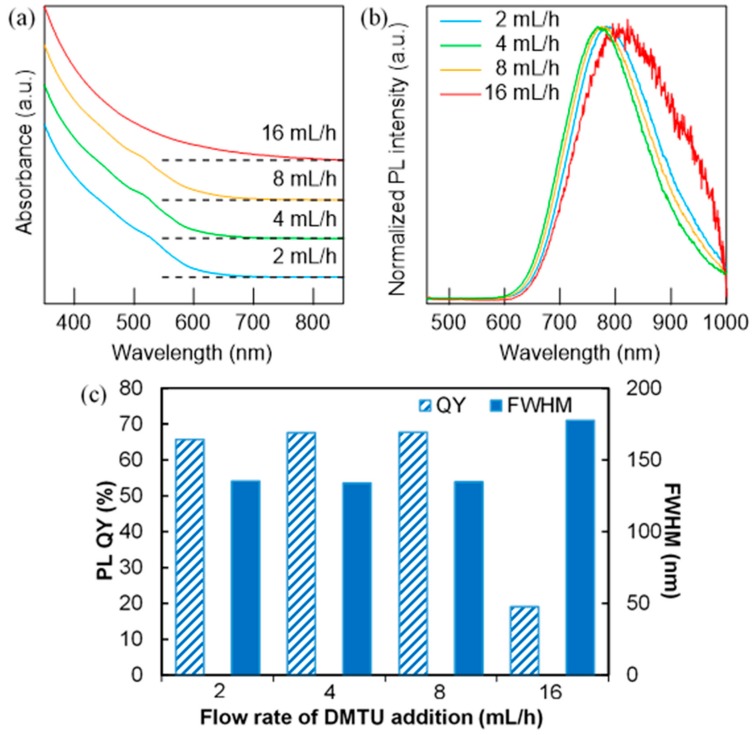
(**a**) UV–Vis absorption and (**b**) PL spectra of AgInS_2_ NPs prepared by the dropwise injection method with a different flow rate of DMTU (2–16 mL/h). (**c**) Comparison of the PL quantum yield (QY) and full width at half maximum (FWHM) of AgInS_2_ NPs prepared at different flow rates of DMTU.

Figure 4a,b show the UV–Vis and PL spectra of AgInS_2_ QDs corresponding to the samples in Figure 3, together with the variation in PL QYs and PL full widths at half maximum (FWHMs) of each sample (Figure 4c). Unlike many previous studies on AgInS_2_ NPs, small excitonic absorption shoulders were observed at ~520 nm for the three samples synthesized using an injection rate smaller than 8 mL/h. This feature disappeared for the sample synthesized at 16 mL/h, and the longer absorption tail emerged (Figure 4a). The appearance of the excitonic peak is generally due to the formation of well-defined NPs with a narrow size distribution and minimal defect sites [31], and these three samples exhibited high PL QYs of over 60%. On the other hand, a distorted broad spectrum with a relatively low PL QY (20%) was obtained for the 16 mL/h sample (Figure 4b), which was mostly comprised of Ag_2_S. These results demonstrate the advantages of the dropwise injection method against the immediate injection method in terms of avoiding the overgrowth of the intermediate Ag_2_S NPs.

**Figure 5:**
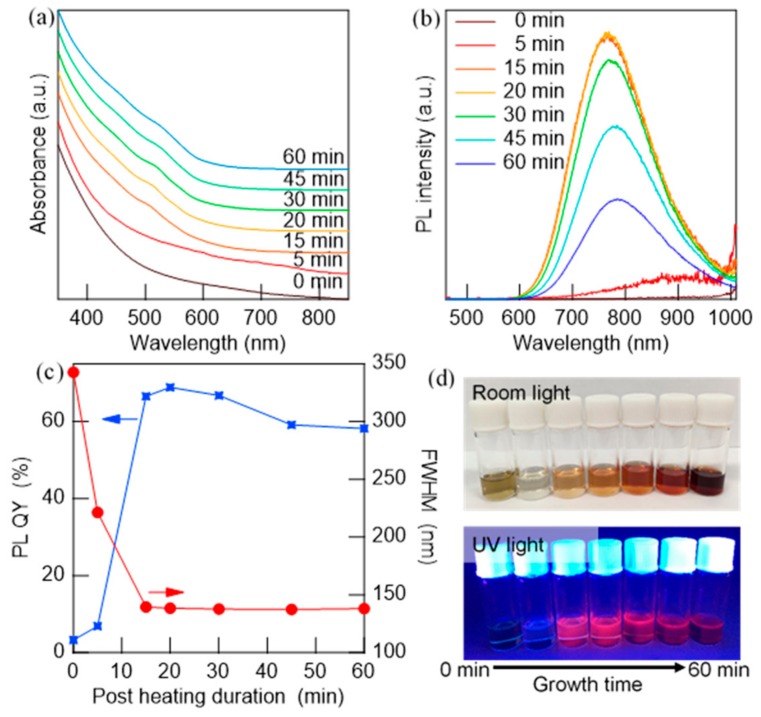
Evolution of (**a**) UV–Vis and (**b**) PL spectra of AgInS_2_ NPs at 140 °C with time after completing the dropwise injection of DMTU (4 mL/h, 0–60 min). (**c**) Change in PL QY values and PL FWHM as a function of growth time (post-heating), and the (**d**) corresponding photographs under room light (**upper**) and UV light (**bottom**).

As above-mentioned, the role of the post-heat treatment is to complete the conversion of Ag_2_S to AgInS_2_. Figure 5a,b show the UV–Vis and PL spectra obtained at different durations of post-heat treatment between 0 and 60 min. The edge of the absorption, which was initially located at >850 nm due to the existence of Ag_2_S NPs [32], blue-shifted with an increase in heating time, and excitonic shoulders at approximately 500 nm became distinct. The NPs were non-luminescent at 0 min (just after the injection was finished), but they started to emit and blue-shifted to 780 nm. The PL QY increased rapidly to 66% after 15 min (Figure 5c), and the absorption tail was similar to that of pure AgInS_2_, and it remained with a slight redshift of the PL (Figure 5b). During heating, after the purification by centrifugation, the color density of the solution considerably changed (Figure 5d) and monotonically increased after 15 min while maintaining a constant absorption profile. Using the adopted reaction conditions, the intermediate Ag_2_S NPs exhibited lower solubility than that of the AgInS_2_ NPs, which was favorable for obtaining a product without complicated purification processes. Therefore, the first increase in solution color density indicates the conversion progress to AgInS_2_ NPs. At the same time, the increase in particle size and change in the crystal phase occurred. These tendencies became more pronounced after 30 min, as shown in the TEM images and size histograms in Figure S3. The average diameter of the NPs increased by approximately 2 nm, with an increase in size distribution (σ ~0.8 nm), which indicates the occurrence of the Ostwald ripening. The XRD pattern shows a change in the crystal phase from tetragonal to orthorhombic during the last 30 min.

### 3.3. Effect of Temperature on the Generation of AgInS_2_ QDs by DMTU Injection

**Figure 6:**
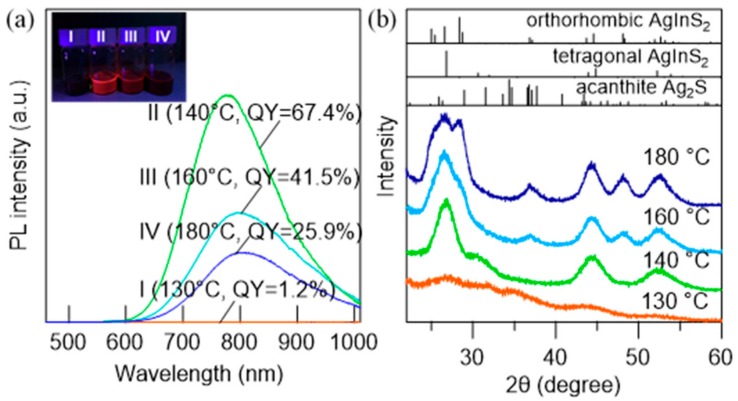
(**a**) PL spectra of AgInS_2_ NPs prepared at different reaction temperatures ranging from 130 to 180 °C using the dropwise injection method (4 mL/h). The PL QYs for each sample are displayed in the same figure. The inset shows photographic images of the corresponding AgInS_2_ NP solutions under UV light. (**b**) XRD patterns of the AgInS_2_ NP samples at different reaction temperatures (130–180 °C), which correspond to the reference patterns of tetragonal-AgInS_2_ (ICSD 077-6632), orthorhombic-AgInS_2_ (ICSD 070-5630), and acanthite Ag_2_S (ICSD 014-0072).

**Figure 7:**
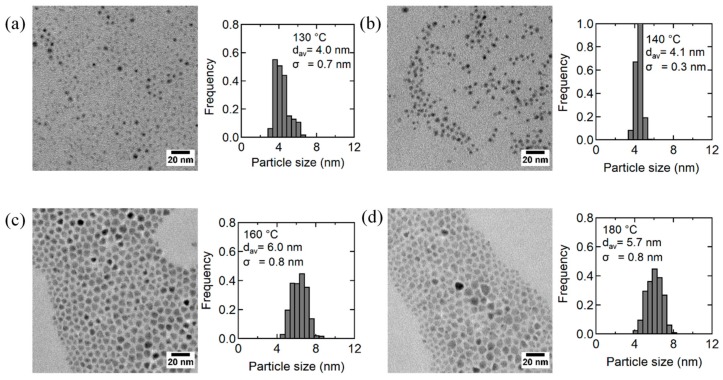
TEM images and the corresponding size distribution histograms for AgInS_2_ NPs prepared by the dropwise injection method (4 mL/h) at different reaction temperatures: (**a**) 130 °C, (**b**) 140 °C, (**c**) 160 °C, and (**d**) 180 °C.

The effects of reaction temperature were investigated in relation to the luminescence performance and morphology of AgInS_2_ QDs. The synthesis was carried out by adding DMTU (flow rate, 4 mL/h) into the metal precursor solution heated at 130–180 °C. In all experiments, the solution temperature was maintained for an additional 30 min after the dropwise injection was completed. Figure 6a shows the PL spectra of the AgInS_2_ QDs synthesized at different reaction temperatures along with the PL QY of each sample. The QDs synthesized at 130 °C did not exhibit PL, which is consistent with the absence of AgInS_2_ features in XRD (Figure 6b). The highest PL QY was obtained at 140 °C, above which the PL QY gradually decreased (Figure 6a and Table 1). In this temperature region, an increase in the particle size and the transformation of the crystal phase from tetragonal to orthorhombic were observed (Figure 6b and Figure 7).

As reported previously, annealing at higher temperatures caused the disordering of cations, which induced the transformation into the orthorhombic phase [19,30]. Figure S4 shows the UV–Vis spectra and the corresponding Tauc plots. The three samples (prepared at 140–180 °C) produced the Tauc plots for a direct semiconductor, which included well-defined straight regions from which the band gap values (*E*_g_) were estimated to be 2.11–2.14 eV. However, the NPs synthesized at 130 °C exhibited an absorption tail at >850 nm, which corresponded to the remaining intermediate Ag_2_S with a band gap of 1.1 eV [33]. The elemental composition of NPs supported similar assumptions (Table 1) that are discussed above, indicating that the less reactive indium species needs a temperature higher than 140 °C to be fully incorporated in nanoparticles. Specifically, the condition of 140 °C with 30 min for post-heating was optimal for synthesizing small tetragonal AgInS_2_ NPs because (i) the formation of Ag_2_S intermediate NPs was sufficiently slow to maintain their small size, and (ii) the conversion from Ag_2_S to AgInS_2_ NPs using indium sulfide clusters was barely possible. The conversion using these cluster species has been reported as a resource–host-type reaction [34]. At higher temperatures, the excessive growth of the intermediate Ag_2_S NPs resulted in an increase in the size of AgInS_2_ NPs, or the Ostwald ripening of the AgInS_2_ NPs generated using this approach may produce unfavorable results [35,36].

### 3.4. Band-Edge Emission by Surface Passivation with Gallium Sulfides

**Figure 8:**
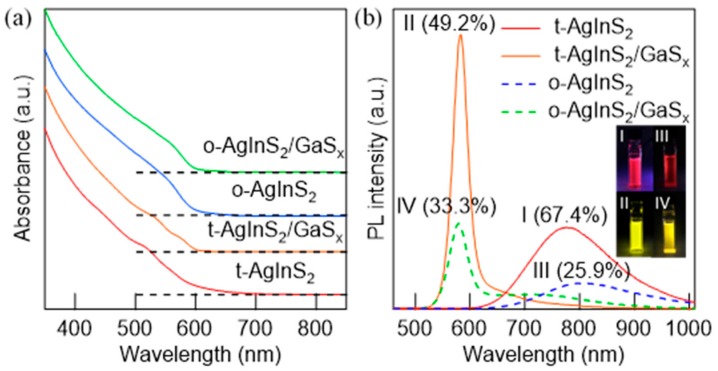
(**a**) UV–Vis absorption and (**b**) PL spectra with displayed PL QYs for the AgInS_2_ cores and AgInS_2_/GaS_x_ core/shell NPs produced at various conditions: (I) tetragonal-AgInS_2_ core (DMTU flow rate = 4 mL/h at 140 °C and post-heating for 30 min), (II) tetragonal-AgInS_2_/GaS_x_ core/shell (prepared from the tetragonal-AgInS_2_ core NPs), (III) orthorhombic-AgInS_2_ core (DMTU flow rate = 4 mL/h at 180 °C and post-heating for 30 min), and (IV) orthorhombic-AgInS_2_/GaS_x_ core/shell (prepared from the orthorhombic-AgInS_2_ core NPs). The inset shows the photographs of the AgInS_2_ core and AgInS_2_/GaS_x_ core/shell NPs in chloroform.

The tetragonal and orthorhombic AgInS_2_ QDs synthesized by the dropwise injection method at different temperatures were coated with gallium sulfide (GaS_x_) shells. The shell formation was achieved using the above-mentioned approach including heating the AgInS_2_ core QDs with the precursors of GaS_x_. Figure 8a shows the UV–Vis spectra for the AgInS_2_ cores and AgInS_2_/GaS_x_ core/shell QDs with different core crystal phases, which are the two types of cores synthesized at 140 °C and 180 °C. The small absorption tail for the tetragonal AgInS_2_ core QDs almost disappeared after they were coated with GaS_x_. Moreover, they showed a slight augmentation of shoulder peaks, which is characteristic of high-quality QDs. On the other hand, the absorption tail for the orthorhombic AgInS_2_ NPs was smaller than that for the tetragonal NPs, and the changes in the absorption after the shell formation were limited. The values of the band gap energies determined from the Tauc plots were 2.17 eV for the tetragonal AgInS_2_/GaS_x_ and 2.14 eV for orthorhombic AgInS_2_/GaS_x_ NPs; these values are the same as those of the original core NPs (Figure S5). The PL spectra for both tetragonal and orthorhombic NPs considerably narrowed after the coating with a GaS_x_ shell, and the PL peak energies (582 nm, 2.13 eV for tetragonal and 578 nm, 2.14 eV for orthorhombic) were consistent with the band gap energies estimated from the Tauc plots. Therefore, these peaks were identical to the band-edge PL (Figure 8b) [27]. The intensity and color purity were different for the two crystal phases. Specifically, the tetragonal-AgInS_2_/GaS_x_ core/shell QDs showed a higher (PL QY = 49.2%) and narrower (FWHM = 32.2 nm) band-edge emission than those of the orthorhombic core/shell QDs (PL QY = 33.3%, FWHM = 37.8 nm). The tetragonal cores clearly produced a more intense band-edge emission than that of the orthorhombic cores. In addition, the values for the tetragonal-AgInS_2_/GaS_x_ core/shell QDs were better than those previously obtained for the same type of the core/shell QDs synthesized using solid thiourea (PL QY = 28.8% and FWHM = 39 nm, without surface treatment by phosphine) [27]. The average values and standard deviations for PL QYs obtained by repeated experiments using three different core synthesis methods are summarized in Figure S6, where the standard deviations (±σ) are represented as error bars. The dropwise method done by the appropriate condition demonstrated the highest QY values both for core QDs and core/shell QDs. The narrower size distribution and well-defined surface structure achieved by the controlled solution-phase reactions resulted in the increase in the PL QYs and narrowing of the PL FWHM. In addition, the PL enhancement by the post-treatment with tri-*n*-octylphosphine remained valid for these samples. The record-high PL QYs of 72.3% and 51.8% were achieved for the tetragonal and orthorhombic samples, respectively, without any noticeable changes in the UV–Vis profiles (Figure S7). Therefore, this enhancement was attributed to the removal of the trap states, which may be present on the surface of the GaS_x_ shells, as we have previously reported [27].

**Figure 9:**
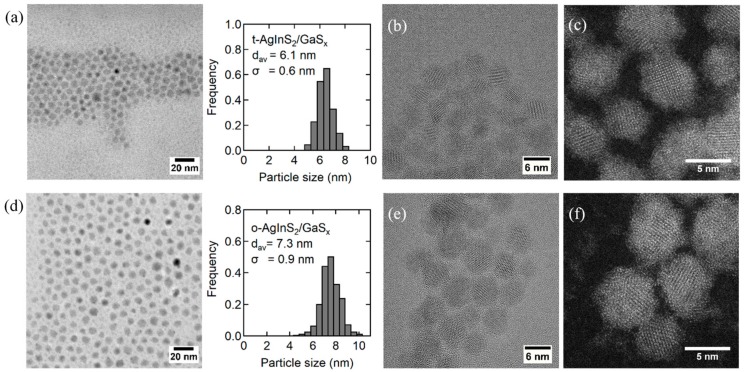
(**a**,**d**) TEM images and the corresponding size distribution histograms, (**b**,**e**) High-resolution TEM (HRTEM) and (**c**,**f**) high-angle annular dark-field scanning transmission electron microscopy (HAADF-STEM) images for AgInS_2_/GaS_x_ core/shell NPs with different crystal structures; (**a**–**c**) tetragonal and (**d**–**f**) orthorhombic.

The TEM observations revealed the morphological changes for the AgInS_2_/GaS_x_ core/shell QDs of two crystal structures (Figure 9) compared with those of the original cores (Figure S8). The EDX spectrum of the same type of nanoparticles for the area, as shown in Figure S9a, clearly indicated the existence of gallium although the sulfur content was slightly less than the expected values due to the partial oxidation and/or photocorrosion of the sample during repetitive purification processes to remove excess ligand that is inevitable to obtain clear TEM images (Figure S9b). The average size of the tetragonal cores increased by 2.0 nm while maintaining a relatively narrow size distribution (σ = 0.6). The orthorhombic cores, which possess a more angular shape and wider size distribution than those of the tetragonal cores, also increased their size by 1.6 nm with σ = 0.9 nm. The HRTEM and HAADF-STEM images of the corresponding core/shell QDs showed clear lattice fringes and well-organized atomic columns, respectively, for the crystalline core portions (Figure 9b,c,e,f). The irregular white dots around the cores in the HAADF-STEM images demonstrated the existence of amorphous shells surrounding the crystalline cores (Figure 9c–f). These results are consistent with the XRD patterns of AgInS_2_/GaS_x_ core/shell NPs, which were almost unchanged after the formation of GaS_x_ shells as shown in Figure S10, indicating the amorphous nature of the shell materials. More accurate composition analyses were performed by using ICP-AES for the purified nanoparticle powders, both before and after coating with GaS_x_, and the results are summarized in Table S2. The Ga:S ratios of 1:0.73 and 1:0.60 for the tetragonal and orthorhombic AgInS_2_ cores, respectively, were obtained under the assumption that the core is stoichiometric, where the ratio of sulfur was similar or slightly smaller than that in our previous report owing to the oxidation of the shell surface. The thicker GaS_x_ shell (1.0 nm) than that in our previous report (0.6 nm) contributed to the increase in the band-edge emission intensity of the tetragonal AgInS_2_ QDs. However, the detailed mechanism of why GaS_x_ shells stick more to the AgInS_2_ QDs prepared with the dropwise injection method needs to be revealed.

**Figure 10:**
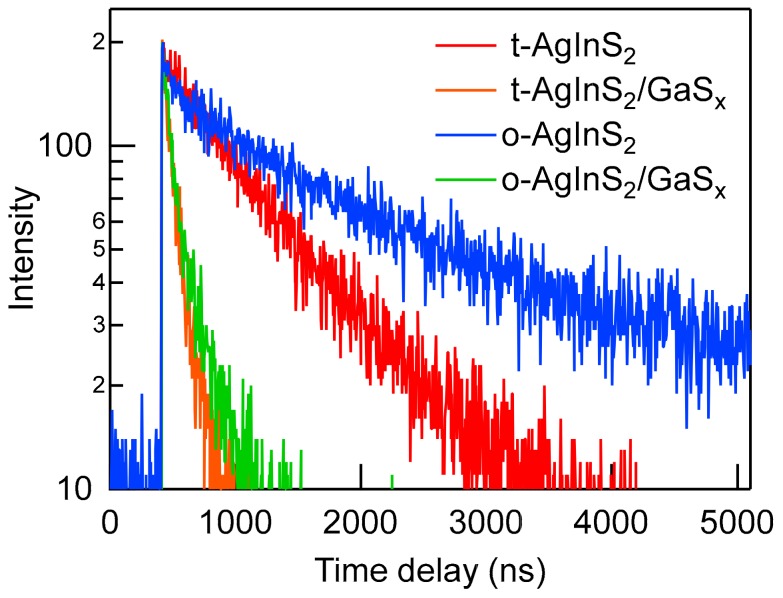
PL decay curves of the AgInS_2_ core and AgInS_2_/GaS_x_ core/shell NPs with two crystal phases for the cores: (red line) tetragonal AgInS_2_ core, (orange line) tetragonal-AgInS_2_/GaS_x_ core/shell (prepared from the tetragonal-AgInS_2_ core), (blue line) orthorhombic-AgInS_2_ core, and (green) orthorhombic-AgInS_2_/GaS_x_ core/shell (prepared from the orthorhombic-AgInS_2_ core). All curves were obtained at the PL peak wavelength of each sample with the excitation at 405 nm.

Figure 10 shows the PL decay curves of the AgInS_2_ core and AgInS_2_/GaS_x_ core/shell NPs with two crystal phases for the core NPs. The PL decay curves for the AgInS_2_ core NPs were fitted using either single or biexponential equations.

(1) $$I\left(t\right)=\underset{n}{\sum }A_{n}\exp\left(-t/\tau_{n}\right)$$

where $I\left(t\right)$ is the intensity at time *t* and $A_{n}$ and $\tau_{n}$ represent the amplitude and decay of each component corresponding to *n*, respectively. The tetragonal AgInS_2_ core NPs can be fit well to a single exponential term with a lifetime of 876 ns (Table 2), which is assignable to the emission from the surface trap-state. The coating of the tetragonal AgInS_2_ NPs with GaS_x_ resulted in a considerable decrease in lifetimes composed of two equally contributing lifetime components of 37.2 ns and 159 ns (as judged by $A_{n}\;\times \;\tau_{n}$) when the decay curve was measured at the wavelength of the newly generated narrow peak. The number of lifetime components required to produce a sufficient curve fitting was smaller than that previously reported [27], which suggests that the NP ensemble of higher uniformity was obtained using the dropwise injection method. In comparison to the tetragonal core NPs, the orthorhombic core NPs had two lifetime components (278 ns and 1976 ns), and 95% of the photons originated from the longer lifetime component. However, when the orthorhombic core NPs were passivated by GaS_x_, the decay components became almost identical to the tetragonal-AgInS_2_/GaS_x_ core/shell NPs, although the crystal structure of the core remained orthorhombic after it was coated with GaS_x_ (Figure S10). We have previously speculated that the longer lifetime component, which is observed for the orthorhombic cores derived from the internal (crystalline) defect levels that cannot be removed by the surface passivation with GaS_x_, and these defect levels decreased the intensity of the band-edge photoluminescence. However, the similarity between the lifetime components of the two crystal phases of the cores after they were coated with the shell evoked a different mechanism. Specifically, the surface of the tetragonal NPs is prone to passivation with GaS_x_ when compared with that of the orthorhombic NPs. Further understanding of the nanostructure interface will help us improve the PL properties of these cadmium-free, ternary semiconductor QDs. This will allow us to produce a replacement for the cadmium-based QDs.

## 4. Conclusions

We have improved the quality of AgInS_2_ core QDs and have successfully enhanced the PL properties of AgInS_2_/GaS_x_ core/shell QDs. By injecting the oleylamine solution of thiourea derivative to the solution containing metal-ions, AgInS_2_ core NPs were synthesized with high monodispersity. As a result of the studies on reaction mechanism, we have demonstrated that the AgInS_2_ NPs were produced through the Ag_2_S intermediate NPs, and the morphology of the AgInS_2_ NPs strongly depended on the generation speed of the Ag_2_S NPs and their conversion to AgInS_2_. After the optimization to obtain small AgInS_2_ core QDs, highly monodisperse (σ = 0.3 nm) AgInS_2_ QDs possessing PL QY of 65% was achieved with high reproducibility. In addition, crystal phases of the AgInS_2_ QDs could perfectly been controlled by changing reaction temperatures. The passivation of the AgInS_2_ QDs with GaS_x_ generated a band-edge emission for both tetragonal and orthorhombic core QDs, with the former crystal phase giving a higher PL QY. Eventually, the tetragonal AgInS_2_/GaS_x_ core/shell QDs emitted band-edge emission of PL QY as high as 72.3% with a narrow FWHM (32 nm) after the passivation of the GaS_x_ shell surface by tri-*n*-octylphosphine. These values are almost equivalent to commercial cadmium-based core/shell QDs, and we expect that the AgInS_2_/GaS_x_ core/shell QDs will replace them in the near future.

## Figures and Tables

**Table 1:** Atomic ratios of the elements and the properties of the AgInS_2_ NPs prepared at different temperatures of 130–180 °C.

Reaction Temperature (°C)	Measured Atomic Ratios			PL QY (%)	FWHM (nm)
	Ag	In	S		
130	1	0.74	2.40	1.2	259.4
140	1	1.11	2.46	67.4	137.1
160	1	1.05	2.30	41.5	143.5
180	1	0.98	2.11	25.9	142.9

**Table 2:** PL lifetime components of the AgInS_2_ core and AgInS_2_/GaS_x_ core/shell NPs corresponding to Figure 10.

NPs ^a^	λ_PL_/nm	τ_1_/ns	A_1_	τ_2_/ns	A_2_	χ^2^
t-AgInS_2_	780	876	1	–	–	1.08
t-AgInS_2_/GaS_x_	582	37.2	0.777	159	0.23	0.98
o-AgInS_2_	803	278	0.29	1976	0.71	1.06
o-AgInS_2_/GaS_x_	578	33.4	0.69	161	0.31	1.00

^a^ t-: tetragonal, o-: orthorhombic.

## Supplementary Materials

The following are available online at https://www.mdpi.com/2079-4991/9/12/1763/s1, Figure S1: EDX spectra of AgInS_2_ QDs; Figure S2: Size distribution histograms of AgInS_2_ prepared at various injection rates; Figure S3: TEM images and the corresponding size distribution histograms of the AgInS_2_ QDs prepared with different annealing time and their XRD patterns; Figure S4: UV–Vis absorption of AgInS_2_ NPs prepared at different reaction temperatures and the corresponding Tauc plots with calculated *E_g_*; Figure S5: Tauc plots with calculated *E_g_* for AgInS_2_ cores and AgInS_2_/GaS_x_ core/shell NPs with different crystal phases of the cores; Figure S6: Average PL QYs of core and core/shell QDs with standard deviation obtained by repeated experiments; Figure S7: UV–Vis and PL spectra of AgInS_2_/GaS_x_ core/shell NPs before and after TOP treatment; Figure S8: TEM images and the corresponding size distribution histograms, HRTEM and HAADF-STEM images for AgInS_2_ core NPs; Figure S9: TEM-EDX spectra of tetragonal AgInS_2_/GaS_x_ core/shell QDs; and Figure S10: XRD patterns of AgInS_2_ core and AgInS_2_/GaS_x_ core/shell NPs. Table S1: Atomic ratios of elements and average diameters of AgInS_2_ QDs; Table S2: Atomic ratios of elements and average diameters for AgInS_2_ cores and AgInS_2_/GaS_x_ core/shell NPs.
