# Supplementary material for: Core Nanoparticle Engineering for Narrower and More Intense Band-Edge Emission from AgInS2/GaSx Core/Shell Quantum Dots
Source: Nanomaterials (Basel). 2019 Dec 11;9(12):1763. doi: 10.3390/nano9121763 (PMC6955825; doi:10.3390/nano9121763)
Supplement: Supplementary file 1 [file nanomaterials-09-01763-s001.pdf]

nanomaterials

MDPI

Article

Core Nanoparticle Engineering for Narrower and More Intense Band-Edge Emission from $AgInS_2/GaS_x$ Core/Shell Quantum Dots

Watcharaporn Hoisang [1], Taro Uematsu [1], Takahisa Yamamoto [2], Tsukasa Torimoto [3] and Susumu Kuwabata [1,*]

[1] Department of Applied Chemistry, Graduate School of Engineering, Osaka University, 2-1 Yamada-oka, Suita, Osaka 565-0871, Japan; hwatcharaporn@chem.eng.osaka-u.ac.jp (W.H.); t-uematsu@chem.eng.osaka-u.ac.jp (T.U.)

[2] Department of Quantum Engineering, Graduate School of Engineering, Nagoya University, Chikusa-ku, Nagoya 464-8603, Japan; yamamoto.takahisa@material.nagoya-u.ac.jp

[3] Department of Crystalline Materials Science, Graduate School of Engineering, Nagoya University, Chikusa-ku, Nagoya 464-8603, Japan; torimoto@chembio.nagoya-u.ac.jp

* Correspondence: kuwabata@chem.eng.osaka-u.ac.jp; Tel.: +81-6-6879-7374

Supplementary material

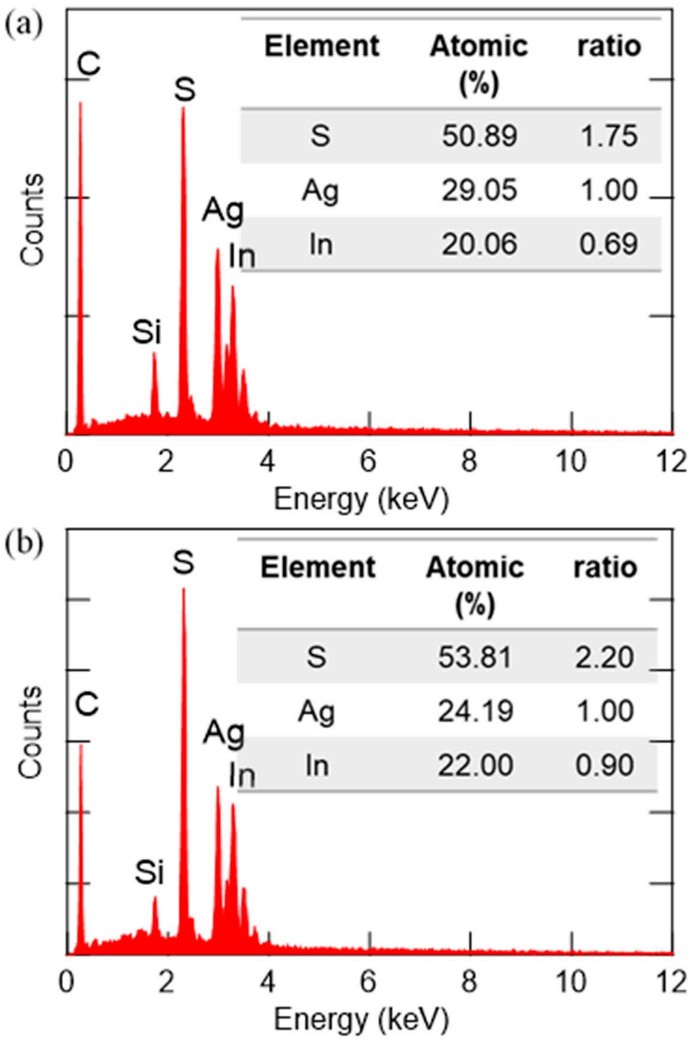

Figure S1. EDX spectra of $AgInS_2$ NPs synthesized by (a) immediate and (b) dropwise injection methods. The inset shows elemental composition of Ag, In, and S. Samples were loaded on a precleaned silicon substrate in the form of chloroform solutions and were dried in air.

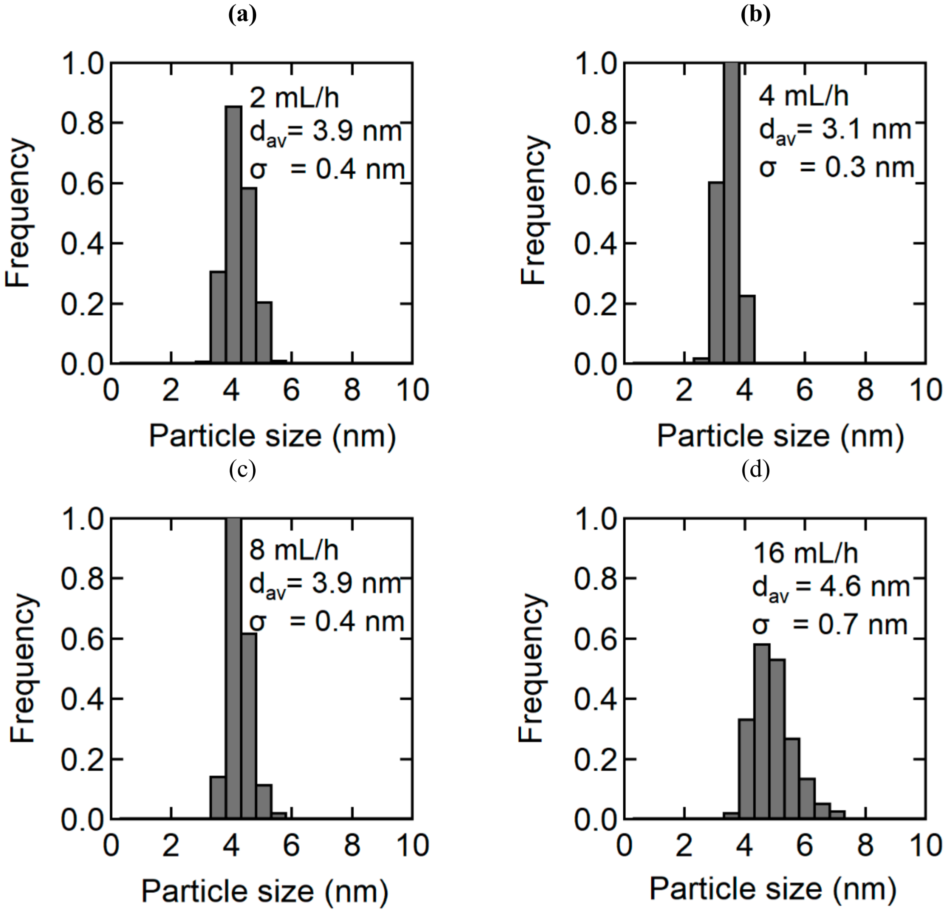

Figure S2. Size distribution histograms of $AgInS_2$ NPs prepared by dropwise injection method at different flow rate of DMTU oleylamine solution: (a) 2 mL/h, (b) 4 mL/h, (c) 8 mL/h, and (d) 16 mL/h. Part of the corresponding TEM images were shown in Fig. 4a–4d.

Table S1. Atomic ratios of elements and average diameters of $AgInS_2$ NPs prepared by dropwise injection at different flow rate of DMTU.

Flow rate (mL/h)	Measured atomic ratio*			Diameter (nm)
	Ag	In	S	
2	1	1.07	2.30	3.9 ± 0.4
4	1	1.07	2.55	3.1 ± 0.3
8	1	1.02	2.30	3.9 ± 0.4
16	1	0.23	1.56	4.6 ± 0.7

*Atomic ratios were measured by ICP-AES.

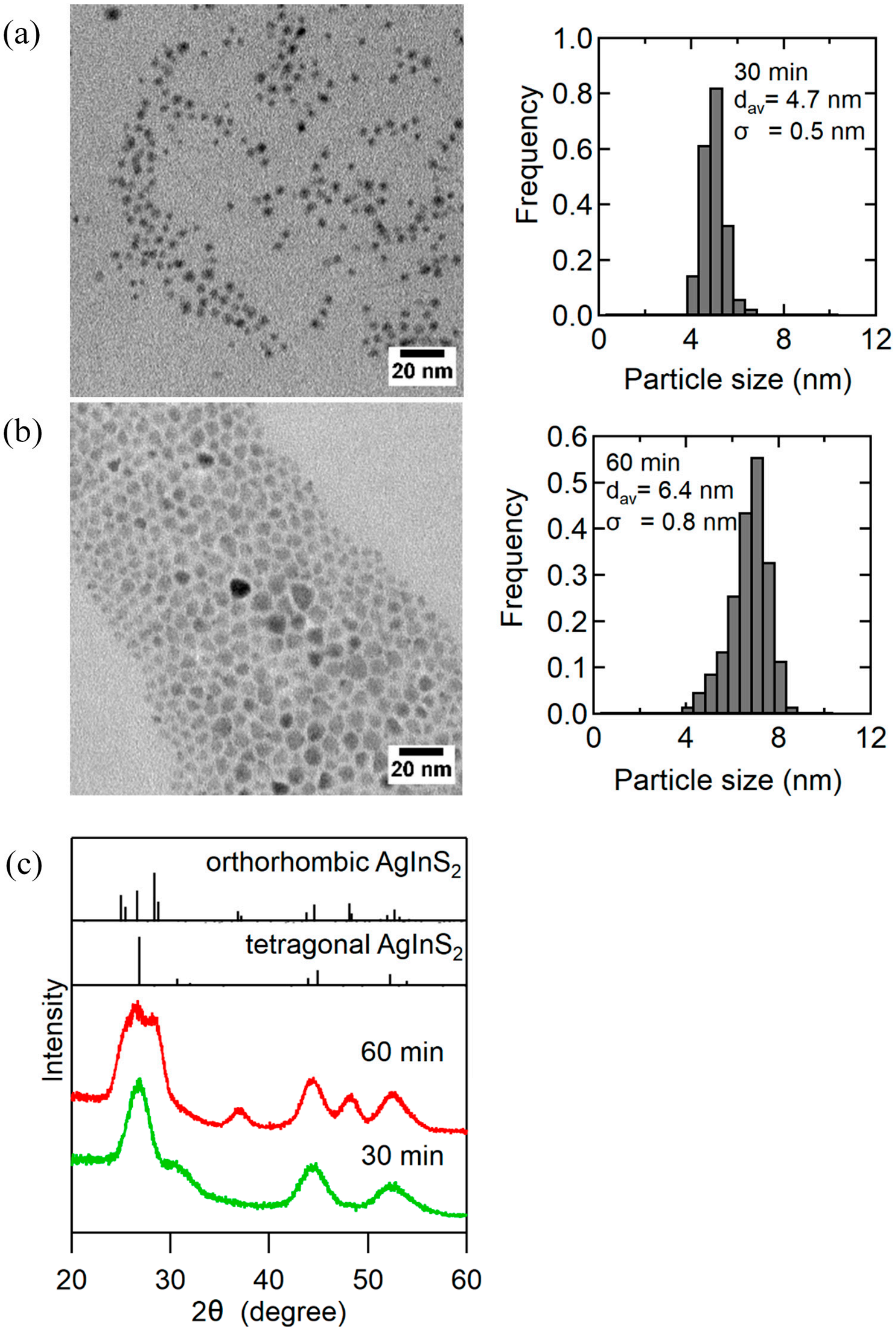

Figure S3. TEM images and the corresponding size distribution histograms of the $AgInS_2$ NPs (a) 30 min, and (b) 60 min after the completion of dropwise injection of DMTU (4 mL/h). (c) XRD patterns of the corresponding samples with reference patterns showing tetragonal-$AgInS_2$ (ICSD 077-6632) and orthorhombic-$AgInS_2$ (ICSD 070-5630).

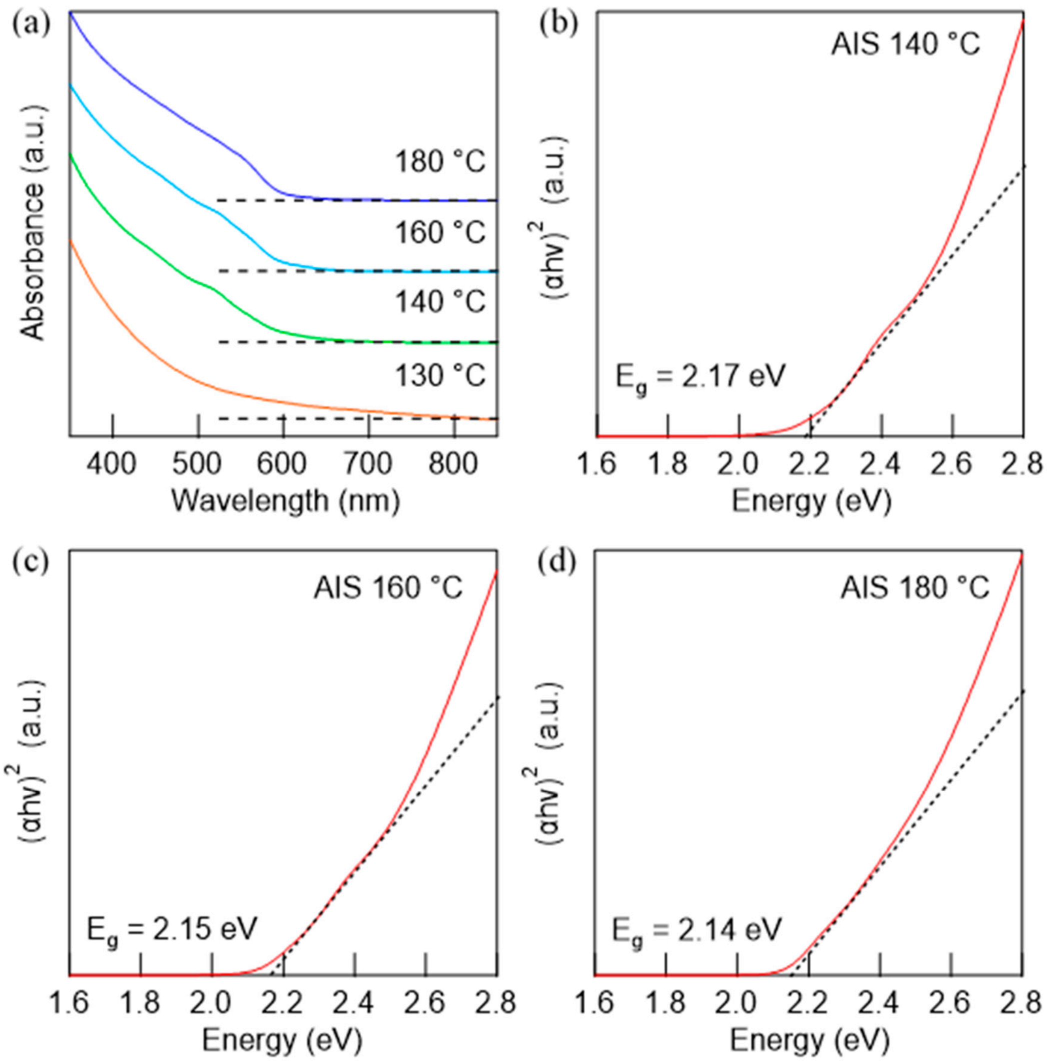

Figure S4. (a) UV-vis absorption of $AgInS_2$ NPs prepared at difference reaction temperatures ranging from 130 to 180 °C using the dropwise injection (4 mL/h). (b)–(d) The corresponding Tauc plots except for the samples of 130 °C with E_g values estimated from the linear regions.

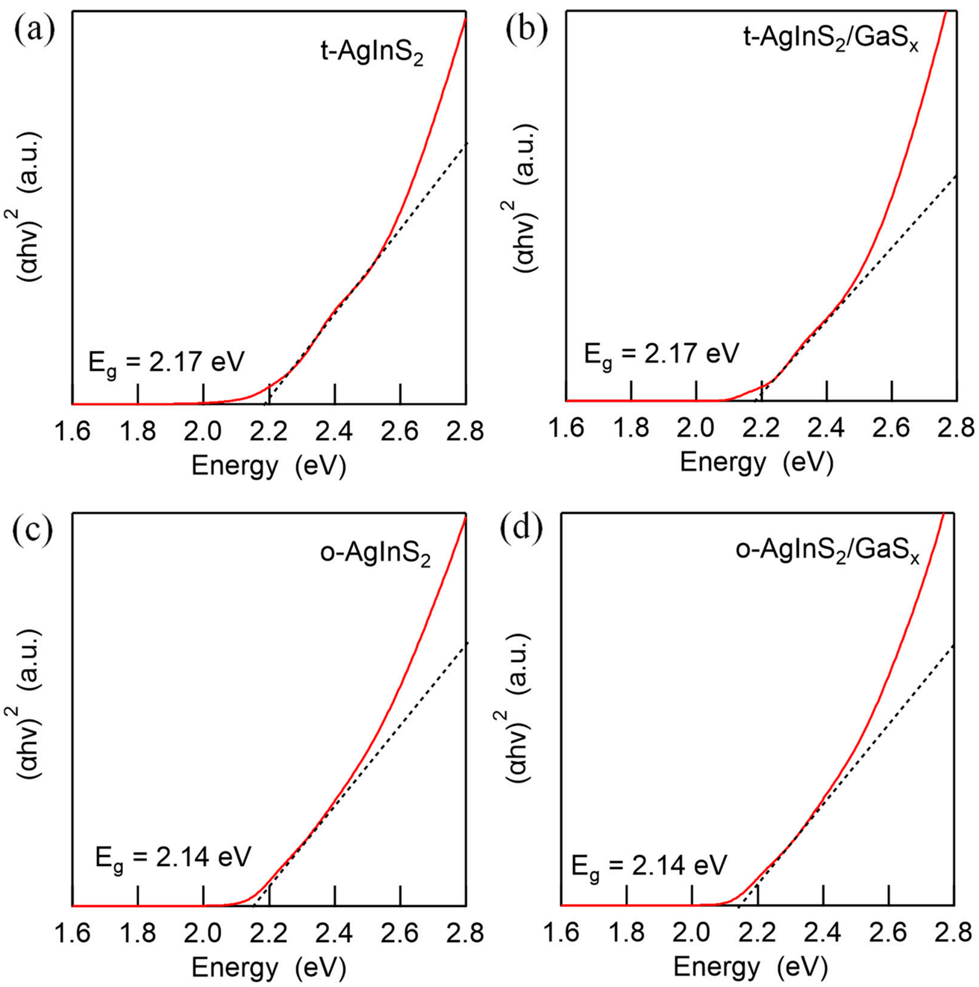

Figure S5. Tauc plots with calculated bandgap energies (E_g) for $AgInS_2$ cores and $AgInS_2/GaS_x$ core/shell NPs having different crystal phases of the cores: (a) t-$AgInS_2$ core NPs (DMTU flow rate = 4 mL/h at 140 °C and post heating for 30 min), (b) t-$AgInS_2/GaS_x$ core/shell NPs (c) o-$AgInS_2$ core NPs (DMTU flow rate = 4 mL/h at 180 °C and post heating for 30 min), and (d) o-$AgInS_2/GaS_x$ core/shell.

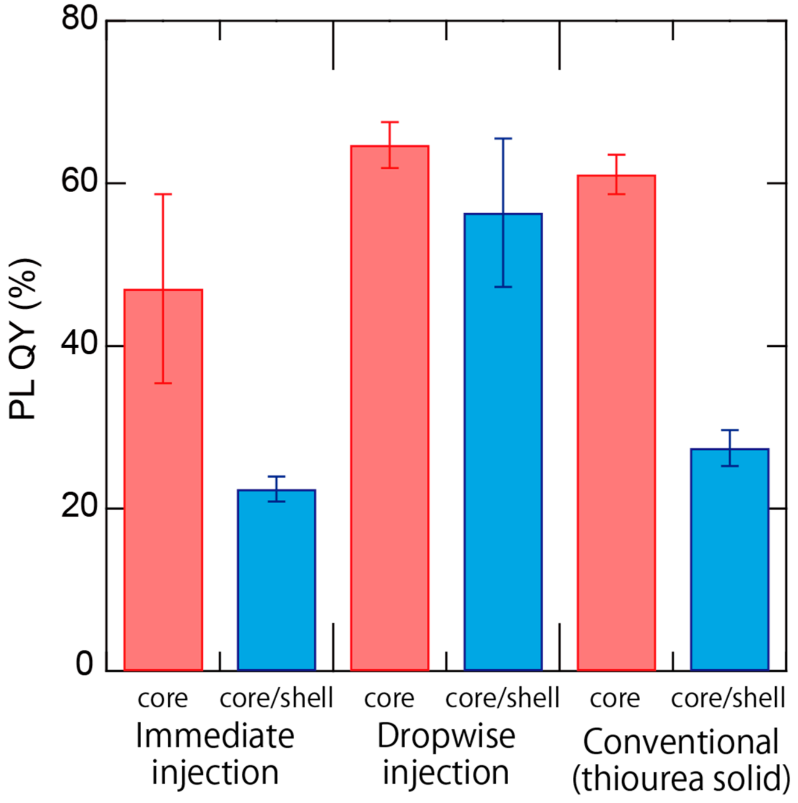

Figure S6. Average PL QY values recorded by repetitive experiments for $AgInS_2$ core and $AgInS_2/GaS_x$ core/shell QDs whose cores were prepared with the three methods including immediated injection, dropwise injection (140 °C, 4 mL/h), and conventional heatup method using solid thiourea. Error bars for each plot are standard deviations (±σ). TOP was not used for all cases.

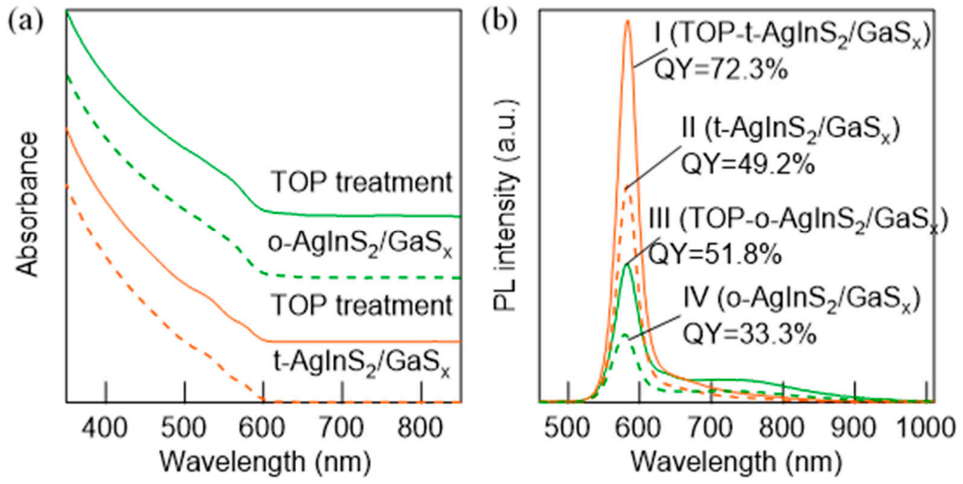

Figure S7. (a) UV-vis and (b) PL spectra of t- and o-$AgInS_2/GaS_x$ core/shell NPs before and after TOP treatment.

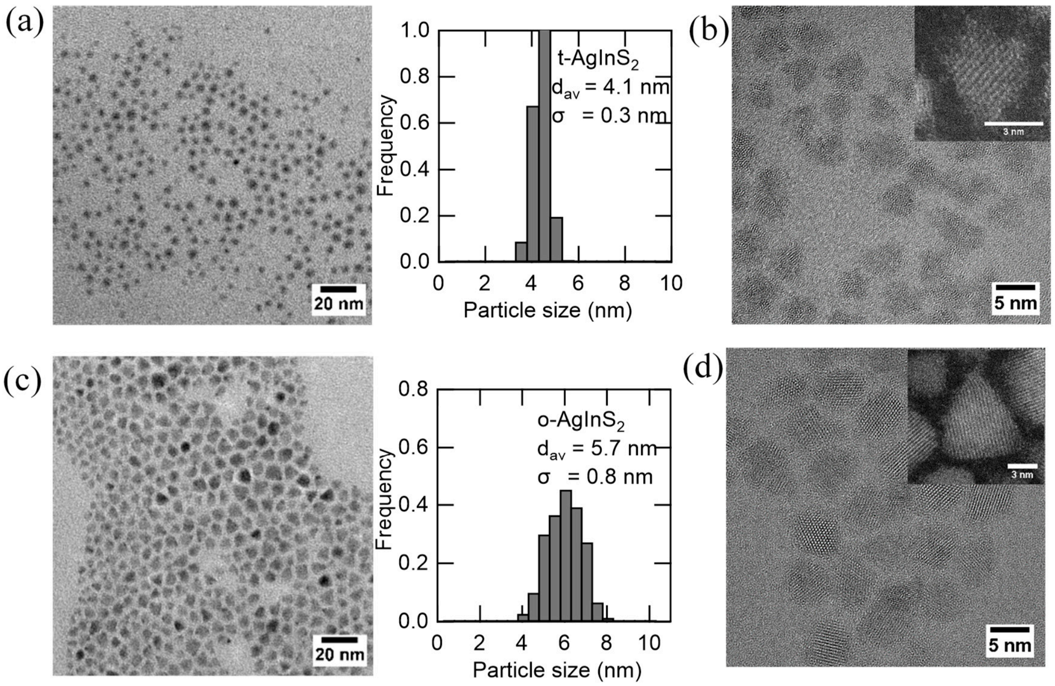

Figure S8. (a, c) TEM images and the corresponding size distribution histograms, and (b, d) HRTEM images for $AgInS_2$ core QDs with different crystal structures; (a, b) tetragonal and (c, d) orthorhombic. Insets are HAADF-STEM images for the both types of QDs.

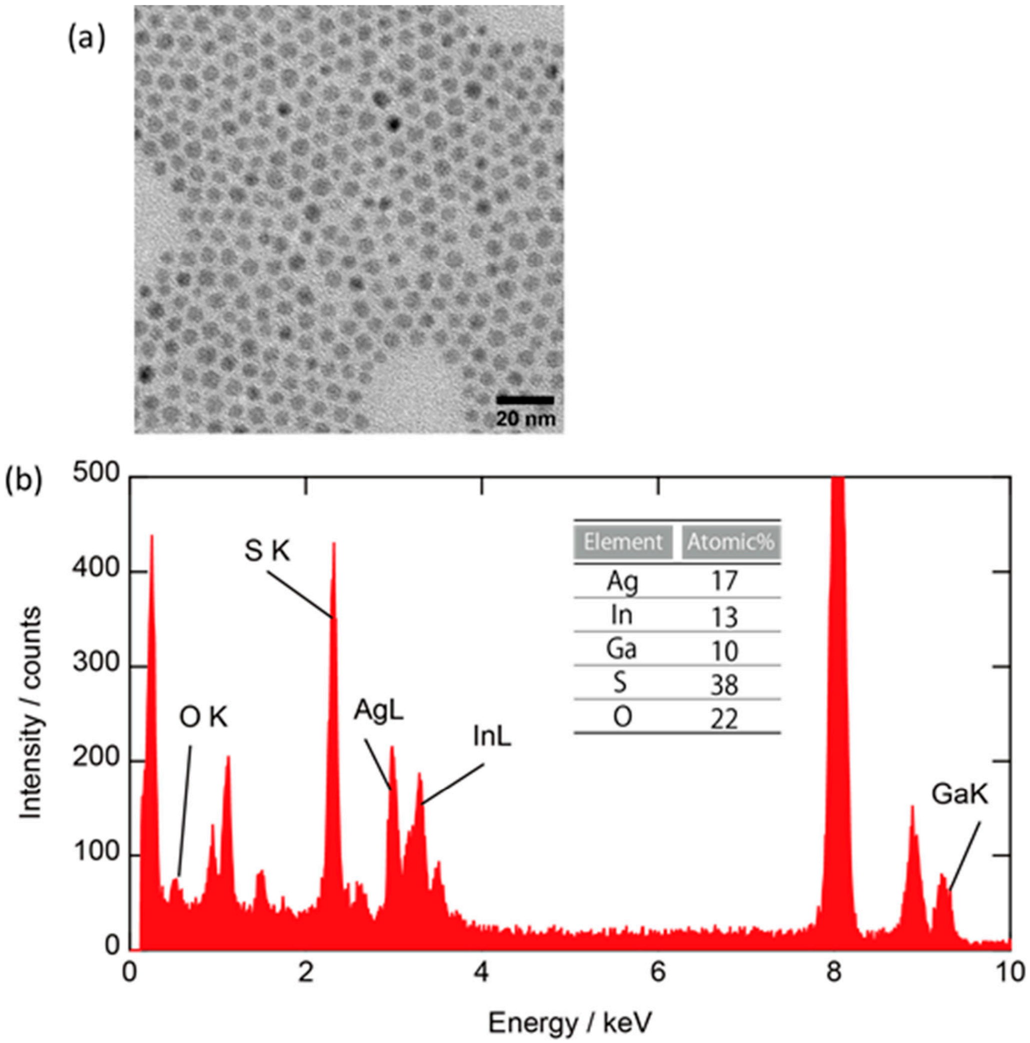

Figure S9. (a) TEM image and (b) EDX (EDAX Genesis equipped with H-7650 TEM) spectrum of the overall area shown in (a). The inset shows composition (atomic %) for the tetragonal $AgInS_2/GaS_x$ core/shell NPs. Other peaks in EDS spectra were assignable either to copper, aluminum, or carbon composing TEM grid and sample holder, and part of small peaks were assignable to other orbitals of these elements. Large part of oxygen should come from the polymer composing TEM grid, but part of them may derive from the nanoparticle sample that is partly oxidized by air.

Table S2. Atomic ratios of elements measured by ICP–AES for $AgInS_2$ core and $AgInS_2/GaS_x$ core/shell NPs with tetragonal and orthorhombic crystal phases of cores.

Sample[a]	Measured atomic ratio (%)				Ga : S ratio	Diameter (nm)
	Ag	In	S	Ga		
t-$AgInS_2$	1	1.11	2.46	-	-	4.1 ± 0.3
t-$AgInS_2/GaS_x$	1	0.85	2.92	1.26	1: 0.73	6.1 ± 0.6
o-$AgInS_2$	1	0.98	2.11	-	-	5.7 ± 0.8
o-$AgInS_2/GaS_x$	1	1.00	4.61	4.33	1: 0.60	7.3 ± 0.9

[a] Ga:S ratios in the shell were presumed by subtracting 2 from the atomic ratios of sulfur.

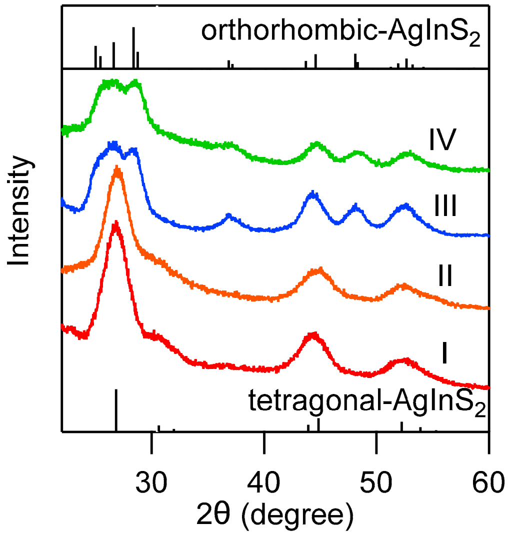

Figure S10. XRD patterns of $AgInS_2$ core and $AgInS_2/GaS_x$ core/shell NPs with various conditions: (I) t-$AgInS_2$ core (DMTU flow rate = 4 mL/h at 140 °C and post heating for 30 min), (II) t-$AgInS_2/GaS_x$ core/shell (prepared from t-$AgInS_2$ core NPs), (III) o-$AgInS_2$ core (DMTU flow rate = 4 mL/h at 180 °C and post heating for 30 min), and (IV) o-$AgInS_2/GaS_x$ core/shell (prepared from o-$AgInS_2$ core NPs). Reference patterns of tetragonal-$AgInS_2$ (ICSD 077-6632) and orthorhombic-$AgInS_2$ (ICSD 070-5630) were shown in the same figure.

© 2019 by the authors. Submitted for possible open access publication under the terms and conditions of the Creative Commons Attribution (CC BY) license (http://creativecommons.org/licenses/by/4.0/).
